# Machine learning applied to whole‐blood RNA‐sequencing data uncovers distinct subsets of patients with systemic lupus erythematosus

**DOI:** 10.1002/cti2.1093

**Published:** 2019-12-12

**Authors:** William A Figgett, Katherine Monaghan, Milica Ng, Monther Alhamdoosh, Eugene Maraskovsky, Nicholas J Wilson, Alberta Y Hoi, Eric F Morand, Fabienne Mackay

**Affiliations:** ^1^ Department of Microbiology and Immunology University of Melbourne at the Peter Doherty Institute for Infection and Immunity Melbourne VIC Australia; ^2^ CSL Limited Parkville VIC Australia; ^3^ Centre for Inflammatory Diseases School of Clinical Sciences Monash University Clayton VIC Australia; ^4^ Department of Immunology and Pathology Central Clinical School Monash University Melbourne VIC Australia

**Keywords:** autoimmunity, RNA‐seq, SLE, stratification, transcriptomics

## Abstract

**Objectives:**

Systemic lupus erythematosus (SLE) is a heterogeneous autoimmune disease that is difficult to treat. There is currently no optimal stratification of patients with SLE, and thus, responses to available treatments are unpredictable. Here, we developed a new stratification scheme for patients with SLE, based on the computational analysis of patients’ whole‐blood transcriptomes.

**Methods:**

We applied machine learning approaches to RNA‐sequencing (RNA‐seq) data sets to stratify patients with SLE into four distinct clusters based on their gene expression profiles. A meta‐analysis on three recently published whole‐blood RNA‐seq data sets was carried out, and an additional similar data set of 30 patients with SLE and 29 healthy donors was incorporated in this study; a total of 161 patients with SLE and 57 healthy donors were analysed.

**Results:**

Examination of SLE clusters, as opposed to unstratified SLE patients, revealed underappreciated differences in the pattern of expression of disease‐related genes relative to clinical presentation. Moreover, gene signatures correlated with flare activity were successfully identified.

**Conclusion:**

Given that SLE disease heterogeneity is a key challenge hindering the design of optimal clinical trials and the adequate management of patients, our approach opens a new possible avenue addressing this limitation via a greater understanding of SLE heterogeneity in humans. Stratification of patients based on gene expression signatures may be a valuable strategy allowing the identification of separate molecular mechanisms underpinning disease in SLE. Further, this approach may have a use in understanding the variability in responsiveness to therapeutics, thereby improving the design of clinical trials and advancing personalised therapy.

## Graphical abstract

We examined whole‐blood RNA‐seq data from a total of 161 patients with systemic lupus erythematosus and 57 healthy donors, uncovering four distinct subtypes of patients with distinct gene expression patterns. Our study provides an innovative patient stratification framework, which is likely to be useful in future studies looking at different responses to treatments, potentially enabling improved therapeutic decisions and more reliable prediction of response to therapy.

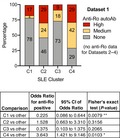

## Introduction

Systemic lupus erythematosus (SLE) is a debilitating chronic autoimmune condition characterised by the activation of inflammatory immune cells and the production of proinflammatory autoantibodies responsible for pathology in multiple organs.^1^ SLE is highly heterogeneous and can be seen as a syndrome rather than a single disease.^2^ The responsiveness of patients to available treatments is variable and difficult to predict. Rather than a small number of highly associated loci, over 60 SLE low‐association loci have been identified by genome‐wide association studies.^3^, ^4^, ^5^, ^6^, ^7^ SLE has been studied using numerous useful mouse models, each of which manifests SLE‐like symptoms underpinned by different molecular mechanisms. Two examples are mice overexpressing B‐cell‐activating factor of the TNF family (BAFF, also known as TNFSF13B), that is BAFF‐transgenic mice, in which low‐affinity self‐reactive B cells aberrantly survive,^8^, ^9^ and glucocorticoid‐induced leucine zipper (GILZ)‐deficient mice^10^ with impaired regulation of activated B cells. These and various other mouse models of SLE replicate some aspects of disease relevant to some patients with SLE, but most likely do not individually account for all the disease symptoms and pathogenesis mechanisms in humans.

Numerous large‐scale clinical trials for SLE treatments have been carried out, with an improvement over standard of care as the expected outcome of these studies. Disappointingly, the vast majority of tested therapies failed their primary endpoints,^11^ except belimumab, an inhibitor of the cytokine BAFF, showing modest efficacy in a subset of patients with SLE.^12^ Highly variable responses to treatments could be explained by the fact that recruitment of patients into clinical trials is based on a limited set of clinical manifestations and/or clinical scores, unlikely to fully capture the differences between patients. Therefore, there is an unmet need for more meaningful patient stratification and recruitment criteria, not just limited to clinical manifestations. Indeed, this can potentially be better achieved using biomarkers reflecting the specific underlying mechanism of disease, allowing for a more mechanism‐targeted and personalised approach to therapy.

Here, we have applied machine learning approaches to stratify patients with SLE based on gene expression patterns derived from whole‐blood transcriptomic data. We demonstrated that this approach identified disease‐linked gene expression patterns not previously visible through conventional data analysis of unstratified patients.

## Results

**Figure 1:**
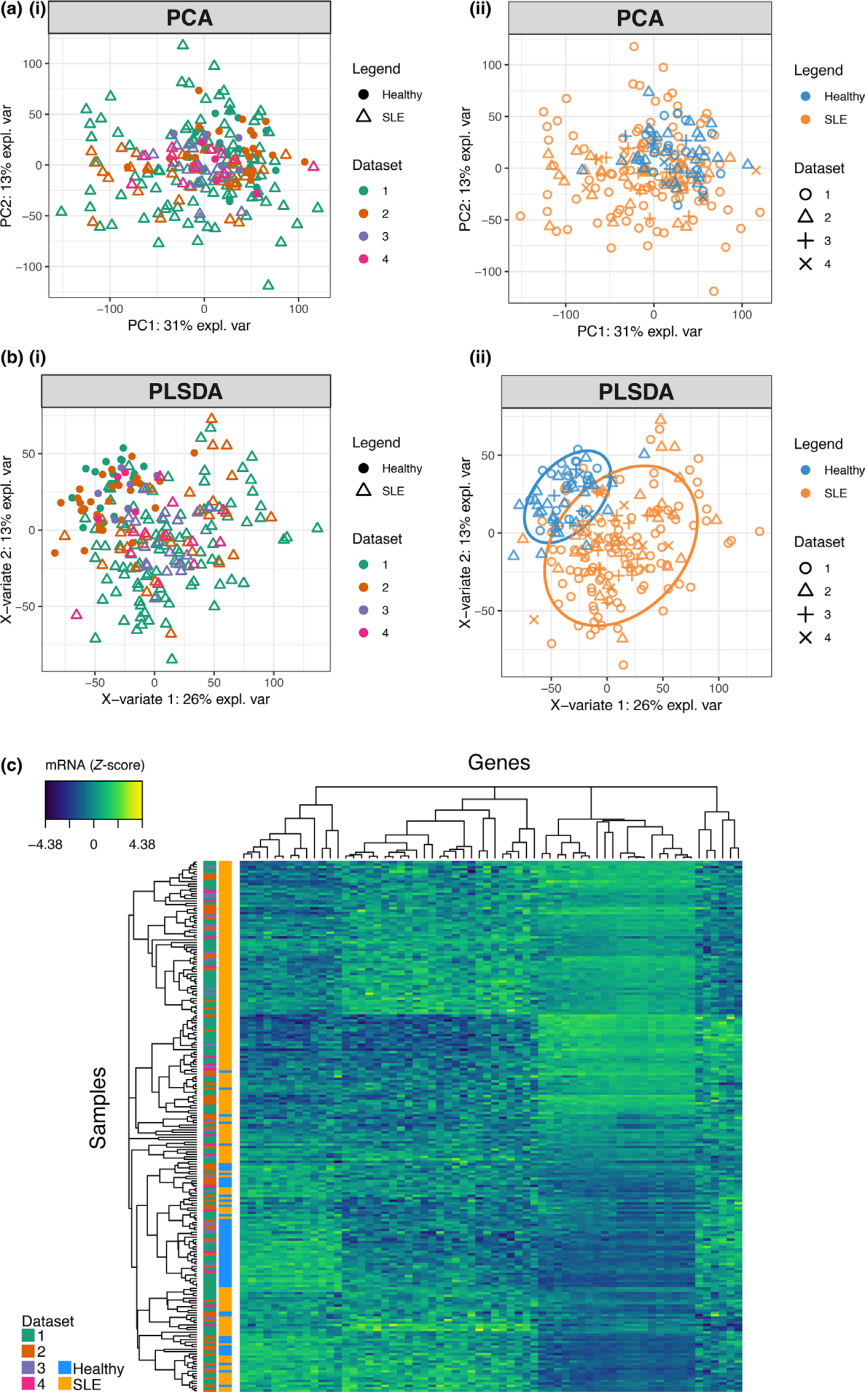
Differential gene expression in SLE. 161 SLE (orange symbols) and 57 healthy donor (blue symbols) transcriptomes from four data sets (see Table 1, shown with different symbol shapes) were examined using multivariate statistics methods. **(a)** Principal components analysis (PCA) was applied to visualise the overall variance between individuals. The same data points are coloured by data set source (left plots) or disease state (right plots) as indicated. **(b)** Partial least squares discriminant analysis (PLSDA), a supervised clustering method, applies weighting to genes, which separate healthy donors and unstratified SLE patients. Ovals indicate the 80% prediction interval. **(c)** Standardised expression levels of top‐weighted genes from the PLSDA model were plotted as a heatmap. Each row is an individual, and each column is a gene.

We examined a cohort of 30 patients with SLE and 29 healthy donors for differentially expressed genes by RNA‐seq, alongside three publicly available independent data sets (161 SLE and 57 healthy donor whole‐blood transcriptomes in total) (Table 1 and Supplementary figure 1).^13^, ^14^, ^15^ Batch effects from combining multiple data sets were taken into account in the differential expression analyses when using limma/edgeR software or otherwise applying ComBat with data set source as a known covariate and verifying a minimal influence of batch effect compared to condition effect using BatchQC (Supplementary figures 1 and 2). Principal components analysis (PCA), which looks at all gene expression and visualises the overall variance between individuals, suggests a higher gene expression heterogeneity in SLE samples than healthy controls, which projected more closely together (Figure 1a). Gene expression in some SLE samples was similar to that of healthy controls. Supervised clustering (to draw apart the groups) was performed using partial least squares discriminant analysis (PLSDA). The PLSDA method assigns greater weighting values to genes that are more useful for separating healthy and SLE patients (Figure 1b). An expression heatmap using the top‐ranking discriminating genes shows heterogeneity across patients with SLE (Figure 1c), but visually demonstrates the possibility of organising SLE patients into several discrete clusters.

**Table 1:** Cohorts of patients and healthy donors, for whole‐blood RNA‐seq data

Data set and reference	Subjects	Collection site	Clinical metadata	RNA‐sequencing method
Data set 1				
Hung *et al.* (2015)^13^ Accession: PRJNA294187	99 SLE (93 female and 6 male)	UCSF Medical Center, USA	Anti‐Ro (‘none’, ‘medium’ and ‘high’) ISM (‘low’ and ‘high’)	Whole blood collected in PAXgene tubes, RNA extracted with TRIzol (Invitrogen, Waltham, MA, USA) RIN checked but not specified TruSeq Library Preparation Kit (Illumina, San Diego, CA, USA) HiSeq 2000 platform (Illumina) 50‐bp SE reads
	18 healthy (female)			
Data set 2				
This study Accession: PRJNA439269	30 SLE (28 female and 2 male)	Monash Medical Centre, Melbourne, Australia	Age Race SLEDAI‐2k, PGA Clinical manifestations Flow cytometry Medications	Whole blood collected in PAXgene tubes, RNA extracted with PAXgene kit (Qiagen, Hilden, Germany) RIN > 7 TruSeq Library Preparation Kit (Illumina) HiSeq 2500 platform (Illumina) 100‐bp SE reads
	29 healthy (27 female and 2 male)			
Data set 3				
Tokuyama *et al.* (2019)^15^ Accession: PRJNA505280	20 SLE 6 healthy	Yale‐New Haven Hospital, USA	Age Race	Whole blood collected in heparin tubes, RNA extracted using RNeasy kit (Qiagen) Library preparation kit for polyA RNA (Illumina) Illumina HiSeq 2500 or NextSeq 500 150‐bp PE reads
	All female			
Data set 4				
Rai *et al.* (2016)^14^ Accession: PRJNA318253	12 SLE 4 healthy	Sir Sunderlal Hospital, Banaras Hindu University, India	Age SLEDAI‐2k Anti‐DNA (±) Anti‐ENA (±) Clinical manifestations Medications	Whole blood collected in heparin tubes, RBC lysis buffer, RNA extracted with TRI reagent (Sigma) RIN > 7 TruSeq Library Preparation Kit (Illumina) HiSeq 2000 platform (Illumina) 100‐bp PE reads
	All female			
Meta‐analysis				
This study. Data sets 1 + 2 + 3 + 4	161 SLE 57 healthy	As above	As above.	As above

All RNA‐seq data are publicly available from the Sequence Read Archive (SRA).^63^ Data sets are numbered in descending order of size. Excluded sample in Data set 2: ‘SLE_21’ (SRR6970317), which was later found to not have SLE.

ENA, extractable nuclear antigens; ISM, interferon signature metric; PE, paired‐end; PGA, Physician Global Assessment; RIN, RNA integrity number; SE, single end; SLE, systemic lupus erythematosus; SLEDAI‐2k, SLE disease activity index 2000; UCSF, University of California, San Francisco.

**Figure 2:**
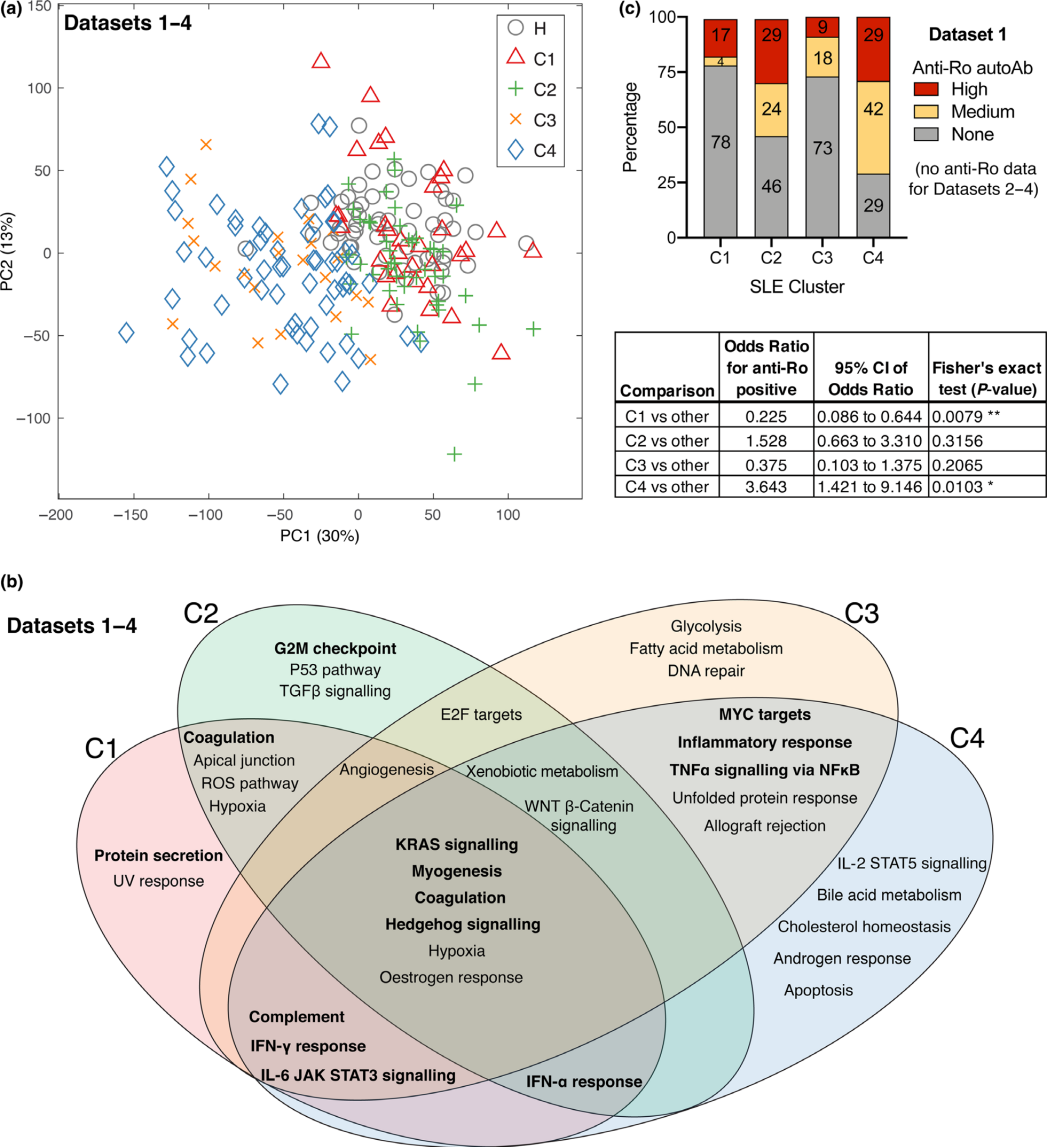
Patient clustering. **(a)** PCA visualisation of 161 SLE whole‐blood transcriptomes after clustering using the *k*‐means algorithm. Four clusters of patients were segregated and displayed with different symbols. Three data sets were combined (see Table 1). **(b)** Venn diagram displaying selected top‐ranking disturbed gene sets (from MSigDB hallmark gene sets) in each SLE cluster C1‐C4 compared to the healthy control group; highest ranking gene sets are bolded. **(c)** Percentage of anti‐Ro autoantibody levels in 99 patients from Data set 1, rated as ‘none’, ‘medium’ or ‘high’, derived from Data set 1 metadata.^13^ The odds ratio of anti‐Ro positivity and Fisher's exact test *P*‐values were calculated for each cluster compared to other patients.

We applied unsupervised *k*‐means clustering to group patients into four clusters, C1‐C4; clusters were visualised with a PCA plot (Figure 2a). The choice of four clusters was based on Gap and Davies–Bouldin clustering evaluations (Supplementary figure 3). The *k*‐means clustering algorithm uses a chosen number of cluster centroids, which are repositioned among the samples until convergence.^16^ We applied PLSDA separately to the two largest Data sets (1 and 2), resulting in similar gene‐weighting values being assigned to draw apart the four clusters, suggesting that this clustering scheme reproduces well in independent study populations (Supplementary figure 4). Supervised machine learning was applied, confirming that classification software can be trained to learn the transcriptomic signatures of each cluster and accurately classify new patients (88% accuracy, Supplementary figures 5 and 6, using two different classifier algorithms).

Cluster 1 (C1) is transcriptionally the most similar to healthy donors, compared to C2‐C4 (Figure 2a). Gene set enrichment analysis was performed to summarise the predominant transcriptomic differences between the clusters (Figure 2b). The top‐ranking disturbed pathways, which differentiate the clusters, include immune activation pathways (e.g. antiviral interferon response), metabolic pathways (e.g. citrate cycle) and DNA repair gene sets. Some of the pathways are likely attributable to particular medications, such as reactive oxygen species (ROS) generation gene sets, which are expressed in response to hydroxychloroquine treatment.^17^

**Figure 3:**
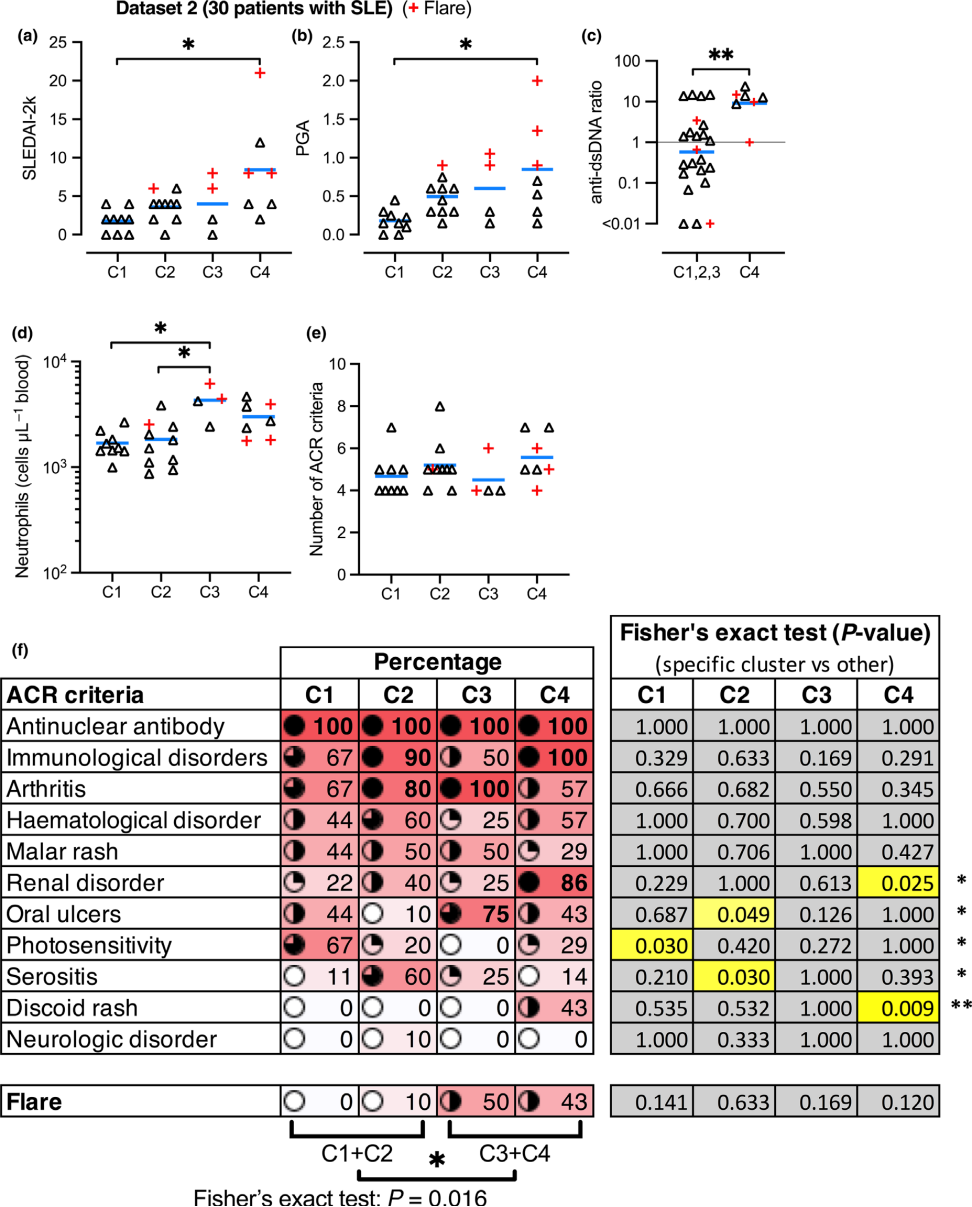
Disease severity and clinical features in SLE subtypes. SLE clusters C1‐C4 in Data set 2 were compared by clinical features. Blue bars represent the mean, and symbols represent patients. Red + symbols represent patients experiencing flares (temporary period of worsened symptoms) at the time of sampling. **(a)** SLE disease activity index 2000 (SLEDAI‐2k). **(b)** Physician Global Assessment (PGA). **(c)** Ratio of anti‐dsDNA autoantibodies, in C4 vs the other clusters combined. **(d)** Circulating neutrophil numbers. **(e)** Total number of ACR criteria each patient was positive for. **(f)** Percentage map of patients in each cluster, who are positive for particular disease features as detailed (ACR criteria) and flare activity.

Interestingly, anti‐Ro autoantibody positivity was increased in C2 and C4; C1 had a significantly decreased anti‐Ro positivity compared to other subsets, whereas C4 had significantly increased anti‐Ro positivity (Figure 2c graph and table with statistics). Ascending levels of overall disease severity were observed from clusters 1 to 4, as suggested by the SLEDAI‐2k (Figure 3a) and Physician Global Assessment (PGA) scores (Figure 3b). Anti‐dsDNA autoantibody ratio was significantly increased in C4 compared to the other clusters (Figure 3c).

Flow cytometry revealed that circulating neutrophil numbers were significantly increased in C3 (Figure 3d). Neutrophils are potentially drivers of nephritis,^18^ but we did not find a significant difference in neutrophil numbers in patients with or without renal disorder in our study population (data not shown). ‘xCell’ (a software tool looking at cell‐specific genes)^19^ calculated enrichment scores, suggesting several significant differences in the representation of some immune cell types in specific clusters (Supplementary figure 7). In particular, the plasma cell gene signature was reduced in C3, whereas B‐cell and CD8^+^ T‐cell gene signatures were reduced in C3 and C4; NKT cell gene signature was increased in C4, while conventional dendritic cell (cDC) gene signature was reduced in C4. M1 and M2 macrophage gene signatures were not significantly altered (Supplementary figure 7).

The 30 patients in Data set 2 all presented with a similar total number of American College of Rheumatology (ACR) criteria (Figure 3e), although there are significant differences in each cluster. For instance, C4 has significantly greater occurrence of renal disorder and discoid rash, whereas C2 has significantly more serositis and less oral ulcers (Figure 3f). C1 has significantly increased occurrence of photosensitivity (Figure 3f). C3 and C4 had significantly more flare activity than C1 and C2 (Figure 3f).

To further investigate the association of gene expression patterns with clinical features, we trained an error‐correcting output codes (ECOC) classifier using the three independent Data sets (1 + 3 + 4), which we then used to classify the patients in Data set 2 (Supplementary figure 8). The predicted clusters reproduced the same clinical distinctions (i.e. increased neutrophils in C3, more disease severity in C4 and more flares in C3 and C4), demonstrating that machine learning may be used as a reliable method detecting differences in clinical features in independent patient cohorts.

**Figure 4:**
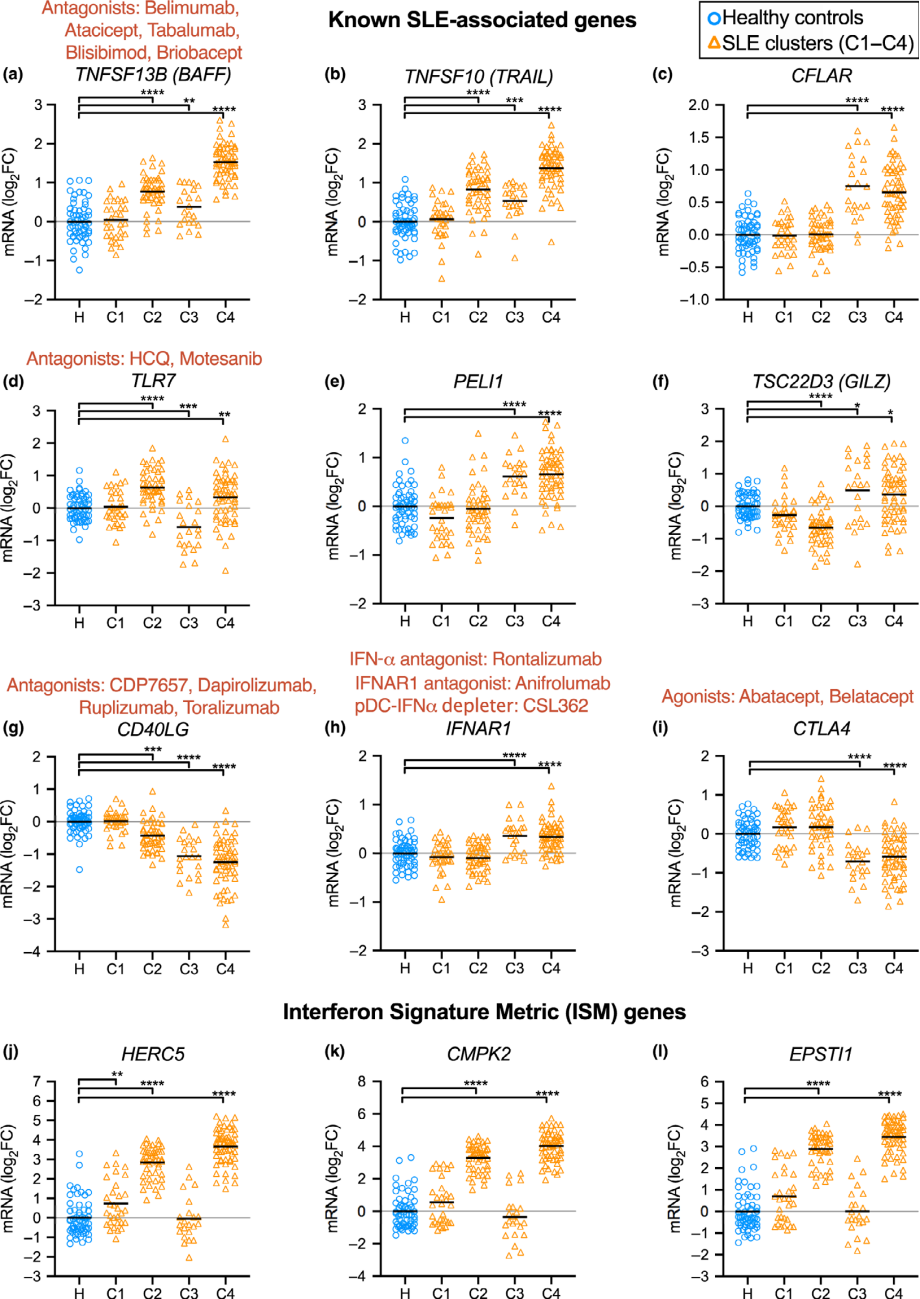
Relative expression levels of known SLE‐associated genes. Expression levels (log_2_ fold‐change relative to the mean of the healthy controls) of **(a)** *TNFSF13B* (*BAFF*), **(b)** *TNFSF10* (*TRAIL*), **(c)** *CFLAR*, **(d)** *TLR7*, **(e)** *PELI1*, **(f)** *TSC22D3* (*GILZ*), **(g)** *CD40LG*, **(h)** *IFNAR1* and **(i)** *CTLA4*. Expression of interferon signature metric (ISM) genes: **(j)** *HERC5*, **(k)** *CMPK2* and **(l)** *EPSTI1*. Therapeutics are indicated in red text above genes coding for the relevant target protein. Three data sets were combined (see Table 1) with batch effects modelled using limma. Significant differences between healthy and SLE samples, using Benjamini–Hochberg‐adjusted *P*‐values, are indicated (*P < 0.05, **P < 0.01, ***P < 0.001 and ****P < 0.0001). Gene expression in unstratified patients is provided in Supplementary figure 9.

In comparing the expression levels of several well‐established SLE‐associated genes in SLE clusters, we found evidence that different pathogenesis pathways may be associated with each cluster of patients (Figure 4), providing more information compared to unstratified analysis (Supplementary figure 9). BAFF (*TNFSF13B*) overexpression is well established as a driver of autoimmunity,^8^ targeted by belimumab. Interestingly, high BAFF expression was a very significant feature of C4 and to a lesser magnitude C2 and C3, but not C1 (Figure 4a). *TNFSF10* mRNA (encoding TNF‐Related Apoptosis‐Inducing Ligand, TRAIL) expression is also upregulated in SLE,^20^ and this mirrored elevated BAFF expression (Figure 4b). Defective apoptosis has been implicated in autoinflammatory settings, including SLE.^21^ Efficient apoptosis can be impaired by upregulation of anti‐apoptotic factors such as cellular FLICE‐inhibitory protein (encoded by *CFLAR*), previously reported to be upregulated in blood B cells of patients with SLE, and correlating with disease severity.^21^ This likely prevents apoptosis signalling in response to ligands such as TRAIL and Fas ligand, to allow aberrant survival of autoreactive cells.^21^ Our stratification found substantial *CFLAR* overexpression in C3 and C4 (Figure 4c).

Excessive TLR receptor signalling is implicated in autoimmunity, with TLR2, TLR7 and TLR9 pursued as potential therapeutic targets in SLE.^22^ Abnormal excessive TLR signalling is thought to exacerbate unspecific immune cell activation.^23^ Interestingly, TLR7 expression was significantly upregulated in C2 and downregulated in C3 (Figure 4d). *PELI1* (encoding Pellino1) is a TLR3‐inducible negative regulator of noncanonical NF‐κB, and the expression of *PELI1* was negatively correlated with disease severity.^24^, ^25^ In our stratification, *PELI1* was not significantly underexpressed in any SLE clusters, but was upregulated in C3 and C4, possibly induced for NF‐κB regulation (Figure 4e). *TSC22D3* (also known as *GILZ*) was identified as a negative regulator of B cells, and lack of *GILZ* drives autoimmune disease (Figure 4e).^10^ *GILZ* expression was markedly diminished in C2, suggesting possible loss of B‐cell regulation. *GILZ* was upregulated in C3 and C4, possibly as an effect of glucocorticoid induction (Figure 4e).

CD40L, encoded by *CD40LG*, mediates T‐cell help driving T‐dependent B‐cell activation and has been unsuccessfully targeted in clinical trials for SLE.^11^ *CD40LG* expression was significantly diminished in clusters C2, C3 and C4, possibly questioning the usefulness of CD40L blockade in those patients (Figure 4g).

*IFNAR1* expression was significantly increased in clusters C3 and C4, suggesting increased interferon signalling sensitivity (Figure 4h). *CTLA4* expression was significantly reduced in C3 and C4, suggesting impaired regulation of effector T cells (Figure 4i). The interferon signature metric (ISM) is a composite score of mRNA expression from three interferon‐regulated genes (*HERC5*, *CMPK2* and *EPSTI1*).^26^ Expression of these genes was consistently upregulated in C2 and C4, whereas C3 levels were comparable to those of healthy donors. Some patients in C1 and some healthy donors had increased levels of ISM genes (Figure 4j–l).

We examined numerous SLE‐associated genes previously identified by GWAS.^3^, ^4^, ^5^, ^6^, ^7^ These genes were significantly differentially expressed in certain clusters, most frequently C4, but also C3 and C2, but not C1 (Supplementary figure 10).

**Figure 5:**
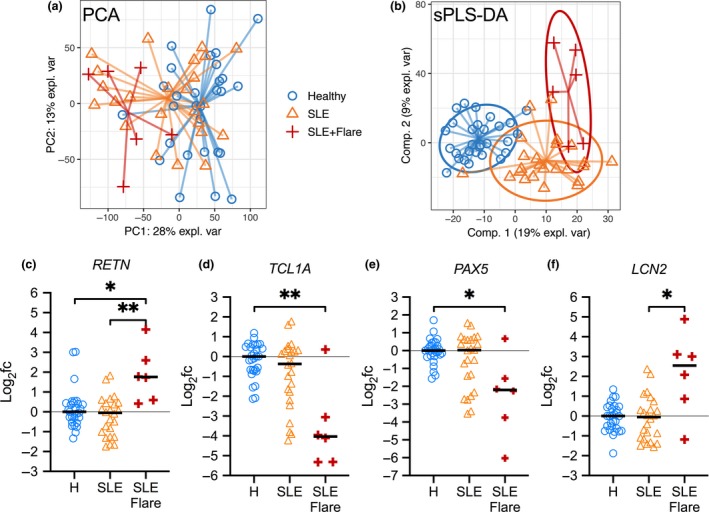
Gene signature for SLE flare activity. Whole‐blood RNA‐seq data from 30 SLE patients (24 without flares and six with flares) and 29 healthy donors were compared (Data set 2, see Table 1). **(a)** Principal components analysis (PCA) to visualise the variation between samples (in all genes); different symbols represent individuals in each group as shown. **(b)** Partial least squares discriminant analysis (PLSDA) was used to select genes that distinguish the groups. **(c–f)** Relative expression of flare‐associated genes, shown as the log_2_ fold‐change relative to the mean of the healthy donor group (‘H’). BH‐adjusted *P*‐values for differential expression (on count data) were calculated using limma (*P < 0.05, **P < 0.01). Gene set enrichment analysis is provided in Supplementary figure 11.

In Data set 2, 6 of the 30 patients with SLE had flares, who diverged further from healthy donors when visualised by PCA (Figure 5a). While numbers are limited, using PLSDA to select flare‐discriminating genes (Figure 5b), we were able to observe differential gene expression during flares consistent with increased innate activation and altered immune cell regulation (Figure 5c–f). Indeed, the *RETN* gene, encoding the proinflammatory adipokine resistin, was upregulated in patients with active flares only (Figure 5c). Resistin is linked to the induction of proinflammatory cytokines.^27^ Significant downregulation of *TCL1A* and *PAX5* (Figure 5d and e) during flares suggests alterations in T‐ and B‐cell homeostasis, respectively.^28^, ^29^ *LCN2* expression was increased in patients with flares (Figure 5f). *LCN2* encodes neutrophil gelatinase‐associated lipocalin (NGAL), which suggests increased neutrophil‐mediated antibacterial activity; NGAL is also a biomarker of kidney injury.^30^ Gene set enrichment analysis of patients with flares suggested increased inflammatory signalling (e.g. IL‐6 and TNF‐α), increased proliferation signalling (KRAS) and haematological disturbances (haem metabolism, coagulation, complement and platelet‐related gene sets) (Supplementary figure 11). These data suggest that our method can be used to look at discrete subpopulations of patients and identify significant differences that can be later validated with larger cohorts.

## Discussion

A universally effective and safe treatment for SLE remains an unmet need because of the heterogeneity of clinical presentations, associated with unpredictable responses to current treatments.^31^ SLE remains a condition with poor long‐term outcome. Over six decades of clinical trials in SLE have only yielded one new therapy, belimumab, an inhibitor of the cytokine BAFF, with mixed efficacy in patients.^11^ Major failures of targeted therapy in the clinic for SLE^11^, ^32^, ^33^ suggest that breakthrough treatments may remain years away. This situation has obligated clinical experts and the pharmaceutical sector to more rigorously understand the reasons for this high failure rate. Suggested factors include issues with the design of clinical trials, difficulty in defining robust endpoints, suboptimal drug targets and biomarkers, study populations that are not broadly representative and high heterogeneity within the study populations.^11^ Large‐scale clinical trials invariably fail to demonstrate efficacy when enrolling patients selected on a limited number of clinical criteria, which do not capture the underlying molecular mechanism likely underpinning disease, which our work showed may vary greatly in patients (Figures 2 and 3). Enrolment of some patients with low disease propensity (C1) further weakens comparisons between placebo and experimental treatment groups.

Our stratification method differentiates patient subgroups with four different gene expression profiles (C1–C4), using whole‐blood transcriptomics to obtain a snapshot of the immune system, and we examined three study populations. This stratification may possibly have a use in improving the design of clinical trials, by more appropriately targeting specific clusters of patients with SLE who possibly express pathology‐relevant genes more homogeneously, suggesting a more consistent mechanism of action underpinning disease in each cluster (Figures 2b and 4). Retrospective analysis of previous failed trials could potentially reveal high efficacy in specific clusters of patients, a possible significant outcome in efficacy currently hidden in unstratified analysis. Successful off‐label usage of rituximab in some patients with SLE further suggests that therapies unsuccessful in clinical trials with SLE may yet have efficacy in selected patients.^34^, ^35^ Indeed, the expression levels of key drug‐targeted molecules such as BAFF and CD40L suggest that certain clusters of patients might be more suitable for the rationale of certain targeted biologics than other clusters (Figure 4). Further studies using RNA samples from patients who participated in clinical trials with differing responses to treatment is the important next step to validate the utility of our method of stratification.

Similar to us, previous studies using microarrays have described distinct clusters of SLE patients in whole‐blood transcriptomic data.^36^, ^37^ Banchereau *et al.* 38 conducted the largest microarray study in SLE, which longitudinally monitored 158 patients with juvenile SLE and uncovered markers associated with disease activity. Recently, Panousis *et al*.^39^ examined 142 patients with SLE and 58 healthy donors by whole‐blood RNA‐seq, and derived additional signals related to global disease activity scores. In this study, we also used RNA‐seq data, which has the advantages of capturing additional genes (not solely restricted to probe sets) and improved dynamic range compared to microarrays. Our study contributes a new stratification scheme derived from the convergence of four RNA‐seq data sets, resolving patients into four main subtypes with machine‐learned gene expression signatures. Additional systems biology approaches (such as microbial metagenomics and metabolomics) are becoming available in SLE, and combining matching data from additional profiling methods may allow for improved sets of clinically useful biomarkers.^40^, ^41^, ^42^, ^43^

Transient flare activity in SLE patients causes a significant surge in inflammation requiring increased medical attention, but much remains to be understood about the underlying molecular mechanism triggering flare activity. We identified several genes that were differentially expressed in patients with flare activity, including the *RETN* gene, encoding the proinflammatory adipokine resistin (Figure 5c). Interestingly, serum resistin levels were elevated in patients with rheumatoid arthritis and/or SLE, although the differences were reported not significant in unstratified patients with SLE, where high heterogeneity was noted.^44^ The specificity of elevated resistin levels to flare‐active patients may explain these results. However, longitudinal studies monitoring successive flares are needed to validate these observations, to identify new flare‐predicting transcriptional signatures and to harness this information for better management of patients with SLE.

The IFN gene signature is a known feature of human SLE, although it does not correlate well with overall disease severity.^26^ Stratification of ISM‐high patients is possible using qPCR assays to monitor expression of three genes in peripheral blood,^26^ which in our stratification corresponded to C2 and C4 (Figures 2b and 4h–l). ISM genes have specificity limitations, shown by a proportion of healthy individuals with elevated levels of these genes, similar to a proportion of C1 patients (who have low disease activity) (Figure 4h–l). Several new treatments related to type I interferon are under investigation, for example anti‐IL‐3Rα (i.e. anti‐CD123 and CSL362 mAb), which depletes basophils and plasmacytoid dendritic cells, cell types that produce type I IFN.^45^ While this treatment may also have therapeutic effects other than that related to limiting type I IFN production, our patient stratification may provide clues as to patients more likely to respond.

In conclusion, our study provides new insights into the heterogeneity of patients with SLE with respect to gene expression in circulating immune cells, which are the messengers of overall immune activity in individual patients. Our novel approach using whole‐blood transcriptomic data combined with machine learning is powerful at segregating and recognising new patient clusters, as well as uncovering cluster‐specific gene expression patterns. Our work is an important first step, examining the underlying genetic heterogeneity of SLE, and our results provide a number of compelling clinically relevant observations, strongly encouraging further validation of our method using future cohorts of patients responding or not to treatments or having or not flares over an extended period of time. Future post hoc analysis of failed clinical trials for SLE using our method may also provide useful information that can help better understand the outcome and refine the design of future clinical trials. As RNA‐seq for each patient is expensive with large cohorts, our work also provides information on cluster‐specific genes, which may be useful when included in new high‐throughput pathology qPCR gene panels identifying clusters, to be further validated. Finally, our in‐depth stratification is potentially the first new opportunity that might put an end to decades of a grim history, plagued with many failures in the clinic in providing patients with SLE with a much‐needed treatment appropriate for the particular subtype of the disease they are suffering from.

## Methods

### Human subjects

Human subjects in Data sets 1 and 3 are previously described (Table 1).^13^, ^14^ Patients with SLE and in Data set 2 were recruited from the Monash Medical Centre.^46^ Healthy donor blood for Data set 2 was collected by the Skin and Cancer Foundation Carlton Victoria after informed consent. Patients with SLE fulfilled the ACR classification criteria.^47^ The SLE disease activity index 2000 (SLEDAI‐2k)^48^ and the Physician Global Assessment (PGA, range: 0–3)^49^ scores were recorded. Blood was collected into PAXgene Blood RNA tubes (BD Biosciences, San Jose, CA, USA), which were frozen at −20°C for later RNA extraction (see below). The titre of anti‐dsDNA autoantibody ratio was calculated using different assays according to the patients' pathology providers (using Farr assay, ELISA and Luminex assay). We have expressed the level of anti‐dsDNA according to the ratio of measured anti‐dsDNA level to the upper limit of normal, and ratio ≤ 1 means normal and not positive for anti‐dsDNA antibodies. Patients did not participate in the analysis.

### RNA extraction and RNA‐sequencing

RNA was extracted using PAXgene Blood RNA Kits (Qiagen). RNA libraries were prepared for sequencing using standard Illumina protocols. RNA‐sequencing (RNA‐seq) was performed on an Illumina HiSeq 2500 platform (all of the samples in cohort 2 were sequenced together); 100‐bp single‐end, stranded reads were analysed with the bcl2fastq 1.8.4 pipeline. Sequence read data are available on Gene Expression Omnibus (GSE112087). Sequencing of the same sample in two lanes showed comparable results (Supplementary figure 10).

### Bioinformatics analysis

#### Read quality, trimming, mapping and summarisation

Publicly available data sets used in this study are listed in Table 1.^13^, ^14^ RNA‐seq data were processed using a consistent workflow (Supplementary figure 1). All software is listed in Supplementary table 1. Read ends were trimmed with Trimmomatic (v0.38) using a sliding window quality filter.^50^ Data sets 2 and 3 were truncated to 50‐bp single‐end format to be consistent with Data set 1, before read mapping. Reads were mapped using HISAT2^51^ (v2.1.0) to the human reference genome GRCh38/hg38, and the GENCODE release v27 of the human genome GRCh38.p10 was used to annotate genes. Read counts were summarised using the *featureCounts* function of the Subread software package (v1.6.1);^52^ nonuniquely mapped reads (i.e. reads that map to more than one gene ambiguously) were excluded from analysis. Males (5% of subjects) were included, but Y chromosome genes were excluded from the analyses. Lowly expressed genes were filtered out using a threshold requiring at least 1 count per million (cpm) in healthy donor samples across all data sets. In total, 9952 genes with unique Entrez accession numbers were retained.

#### Normalisation, standardisation and batch analysis

Read counts were normalised by the upper‐quartile method, to correct for differences in sequencing depth between samples, using edgeR.^53^, ^54^ Counts were log_2_‐transformed with an offset of 1, and samples in each data set were computed as the log_2_ fold‐change (log_2_fc) against the matching healthy control group mean. These processing steps were useful to reduce the distracting effects of extreme values and skewness typically found in RNA‐seq data.^55^ Batch effects (expected when combining data sets) were taken into account in the statistical models using limma/edgeR for differential gene expression testing (see below), or reduced using data set source as a known covariate using ComBat and verified using BatchQC (Supplementary figure 2).

#### Gene selection, clustering and machine learning

Principal components analysis and PLSDA were performed using the mixOmics R package (using Lasso penalisation to rank predictive genes)^56^ and the MUVR R package (v.0.0.971).^57^ Cross‐validation was used to protect against overfitting: in mixOmics, using M‐fold cross‐validation (10‐fold averaged 50 times); and in MUVR, using 15 repetitions of repeated double cross‐validation. A repeated‐measures design was used when combining data sets.^58^ Unsupervised clustering was performed with MATLAB (MathWorks, Natick, MA, USA), using the *k*‐means function (using 100 repetitions to optimise initial centroid positions). The number of clusters was chosen based on unsupervised hierarchical clustering with MATLAB. ECOC classifiers, which contain several support vector machines for multiclass identification, were generated using MATLAB. Random forest classifiers were generated using MUVR.^57^

### Differential gene expression and gene set enrichment analysis

#### Count‐based expression analyses

The limma/edgeR workflow was used for differential expression analysis, considering each data set as a batch.^54^ The EGSEA (v1.10.1) R package was used to statistically test for enrichment of gene expression sets, using a consensus of several gene set enrichment analysis tools.^59^ EGSEA uses count data transformed with *voom* (a function of the limma package).^60^ Collections of predefined gene sets were from KEGG Pathways and the Molecular Signatures Database (MSigDB: ‘h’ hallmark and ‘c2’ curated collections).^61^

### Circulating immune cell composition analysis

#### Flow cytometry

Whole‐blood samples collected into lithium heparin tubes (BD) were examined for frequency of circulating neutrophils (SSC^high^ CD11b^+^, CD49d^−^) by flow cytometry. Whole‐blood samples were stained for 15 min at room temperature before being fixed with BD lysing solution (BD) and acquired on a MACSQuant 10 (Miltenyi Biotec, Bergisch Gladbach, Germany) with subsequent analysis done with FlowJo software (Tree Star, Ashland, OR, USA).

#### Transcript‐length‐adjusted expression and cell‐type enrichment analysis

Transcript‐length‐adjusted expression estimates (FPKM, Fragments Per Kilobase of transcript per Million mapped reads) were obtained using StringTie (v1.3.4) and Ballgown (v2.12.0) R packages.^51^ Whole‐blood RNA‐seq results (FPKM format) were analysed for immune cell‐type signature enrichment using the xCell R package (v1.1.0).^19^

### Statistical analysis

The mixOmics and MUVR R packages were used for multivariate analysis using count data.^62^ The limma R package was used to test for significantly differentially expressed genes while modelling batch effects (expected from combining data sets) and correcting for multiple comparisons (i.e. testing thousands of genes) using the Benjamini–Hochberg procedure. R version 3.5.2 was used. Fisher's exact tests and odds ratio calculations on contingency table data were performed using Prism software (v8.0.2; GraphPad Software, San Diego, CA, USA). Statistically significant differences are shown for *P* < 0.05 (*), *P* < 0.01 (**), *P* < 0.001 (***), *P* < 0.0001 (****) or not significant (n.s.).

## Authors’ contributions

WAF conducted the analysis, wrote source code, produced the figures and wrote the manuscript. FM, EFM, KM, MA, MN and NJW reviewed the manuscript. KM, MN, MA, EFM, NJW, AYH and EFM generated Data set 2.

## Conflict of interest

KM, MN, MA, EM and NJW are employees of CSL Ltd.

## Patient consent

Written informed consent was obtained from all subjects.

## Ethics approval

Ethics approval was obtained from the Human Research Ethics Committee at Monash Health.
