# Supplementary material for: Machine learning applied to whole‐blood RNA‐sequencing data uncovers distinct subsets of patients with systemic lupus erythematosus
Source: Clin Transl Immunology. 2019 Dec 12;8(12):e01093. doi: 10.1002/cti2.1093 (PMC6946916; doi:10.1002/cti2.1093)
Supplement: Supplementary file 1 [file CTI2-8-e01093-s001.pdf]

Supplementary material

Supplementary figure 1. Bioinformatics workflow. Four RNA-seq datasets (Table 1) were processed consistently using the depicted workflow.

Supplementary figure 2. BatchQC. Four RNA-seq datasets (Table 1) were batch-corrected using ComBat and assessed for remaining batch contribution using BatchQC software.

Supplementary figure 3. *k*-means cluster number scoring. 161 patients with SLE, from four RNA-seq datasets (Supplementary table S1), were assessed for optimal cluster number by Gap evaluation (**a**), and by Davies-Bouldin evaluation (**b**). These evaluations support stratification into four clusters of patients.

Supplementary figure 4. Cluster-discriminating gene expression patterns in independent SLE populations. RNA-seq data of 99 patients with SLE from Dataset 1 (**a**), and 30 SLE patients from Dataset 2 (**b**) were separately analysed by partial least squares discriminant analysis (PLSDA). (**c-d**) The eigengene component weightings from each dataset PLSDA were compared to assess if similar genes were being used to draw apart the four clusters (C1-C4) in these independent study populations. The red line shows the trend and blue lines show the 95% prediction interval.

Supplementary figure 5. SLE subset discrimination using support vector machine classifiers. An error-correcting output codes (ECOC) classifier was trained using Dataset 1, to learn how to distinguish SLE clusters (C1-C4); in this case healthy donors were grouped with C1. The accuracy of the classifier was tested using independent cases from Datasets 2+4, checking whether the cluster identification matches the original clustering by *k*-means in figure 2.

Supplementary figure 6. SLE subset discrimination using random forest classifiers. Three whole-blood RNA-seq datasets encompassing 141 patients with SLE were clustered (as in figure 1). (**a**) Random forest classifiers were trained and tested using repeated double cross validation to protect from overfitting, while selecting optimal gene sets with predictive value, using the minimum number of genes ('min'), maximum number of genes with predictive value ('max'), or the geometric mean from those models ('mid'). Using very few genes (on the left of

this plot) results in a higher error rate; using too many genes with no added predictive value (on the right side of this plot) also results in a higher error rate due to the accumulation of noise. (**b**) Performance testing of the 'mid' classification model, which had 88% overall accuracy to predict the original cluster type, using 49 genes. For each sample (each horizontal lane), the predicted probability of each cluster designation (coloured symbols) is plotted. Incorrect classifications are circled. Smaller symbols show the result of test repetitions, larger symbols show the average result from the repetitions.

Supplementary figure 7. Cell subset deconvolution. 30 patients with SLE patients from Dataset 2 were stratified into four clusters (C1-C4); patients with flares are indicated with red (+) symbols. Blue bars show the mean. Immune cell type enrichment in whole-blood RNA-seq data was estimated from FPKM values using xCell.[19] Signature enrichment scores for: (**a**) B cells and plasma cells; (**b**) CD8+ T cells, natural killer T cells (NKT); (**c**) conventional dendritic cells (cDC), M1 macrophages, M2 macrophages. Statistically significance was assessed using Kruskall-Wallis tests, with Dunn's correction for multiple comparisons.

Supplementary figure 8. Cluster validation by clinical features. (**a**) Datasets 1+3+4 (collectively 131 patients with SLE) were used to train an error-correcting output codes (ECOC) support vector machine classifier to recognise four clusters of patients with SLE (C1-C4), then the classifier was used on patients in Dataset 2 (30 patients with SLE). (**b**) SLEDAI-2k disease activity. Patients with flares are indicated with a red '+' symbol. (**c**) Circulating neutrophil cell numbers.

Supplementary figure 9. Expression of known SLE-associated genes in unstratified patients. 161 patients with SLE and 57 healthy controls, from four Datasets, were examined for mRNA expression of known SLE-associated genes by RNA-seq. Gene expression is expressed as the log2-FC relative to the mean of healthy donor controls. Black bars show the means. This data relates to Figure 4.

Supplementary figure 10. Differential expression of GWAS-identified genes. 161 patients with SLE and 57 healthy controls from four datasets (Table 1) were examined for differential expression of 16 SLE-associated genes identified from GWAS studies (in order of best *P*-value given by GWAS Catalog).[7] Gene expression is expressed as the log2-FC relative to the mean of healthy donor controls. Black bars show the means.

Supplementary figure 11. Gene set analysis of patients with flares. 30 patients with SLE from Dataset 2 were divided into patients with active flares (n=6) or not (n=24) and examined for gene set enrichment using EGSEA software. The top-ranking disturbed pathways are displayed in order of statistical significance.

Supplementary figure 12. Sequencing lane replication. RNA libraries in this study (Dataset 2), were sequenced on a second lane with very consistent results, visualised by principal coordinates analysis (PCA).

Supplementary figure 1

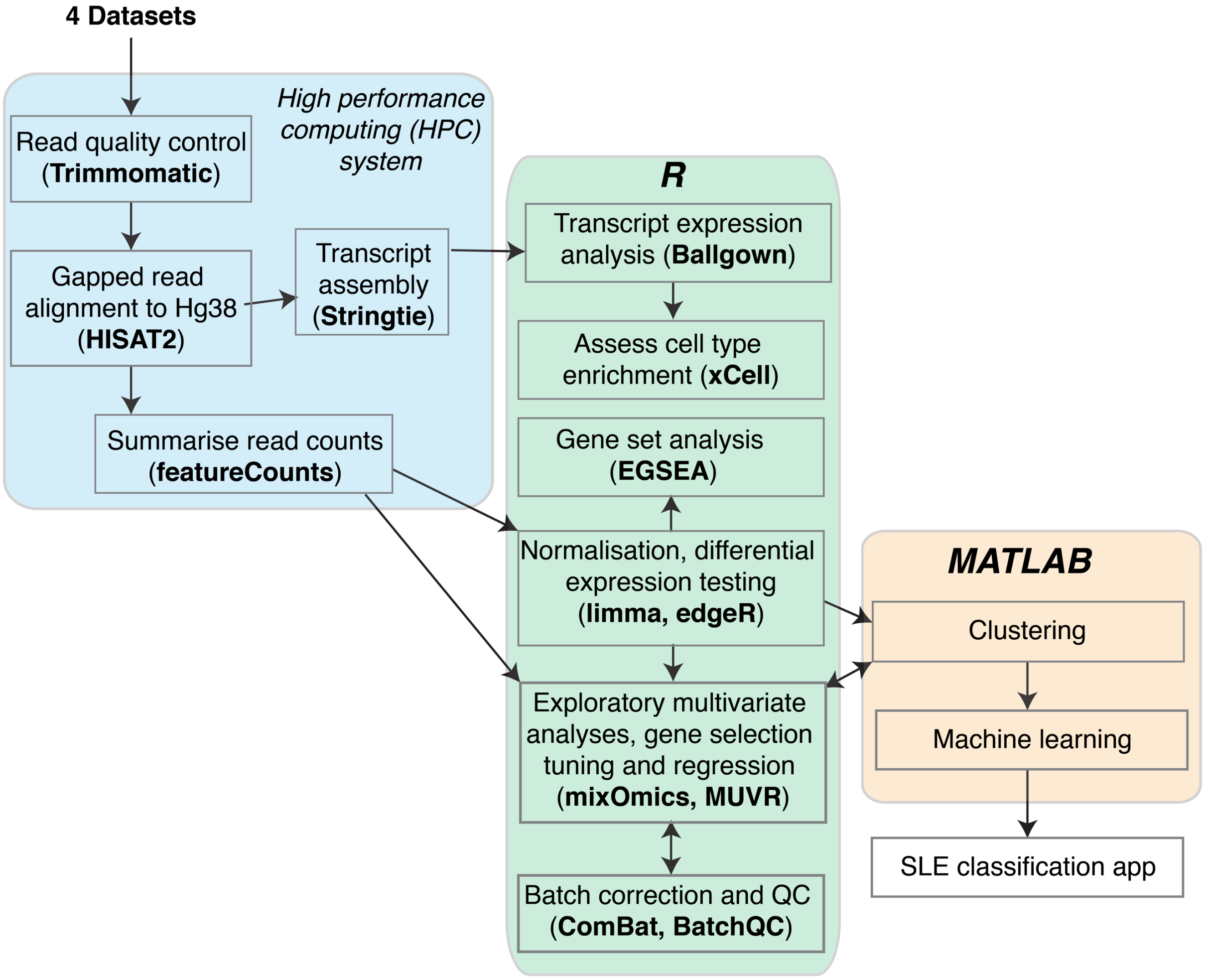

Supplementary figure 2

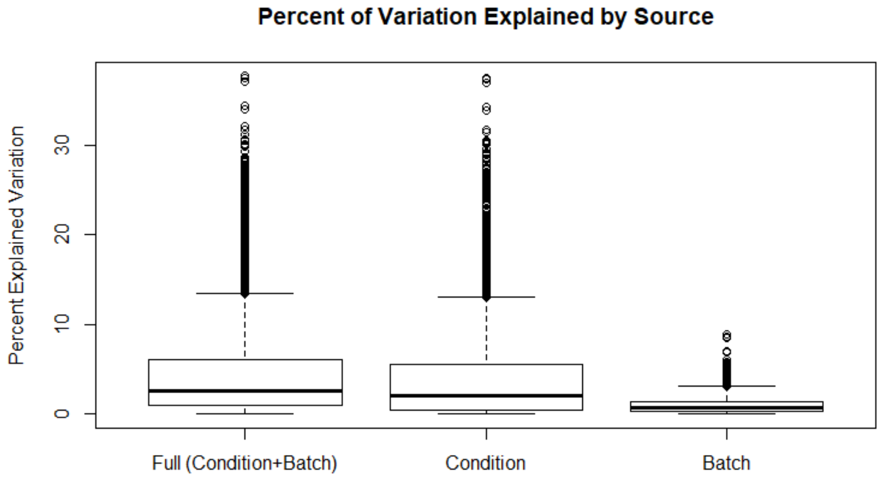

Supplementary figure 3

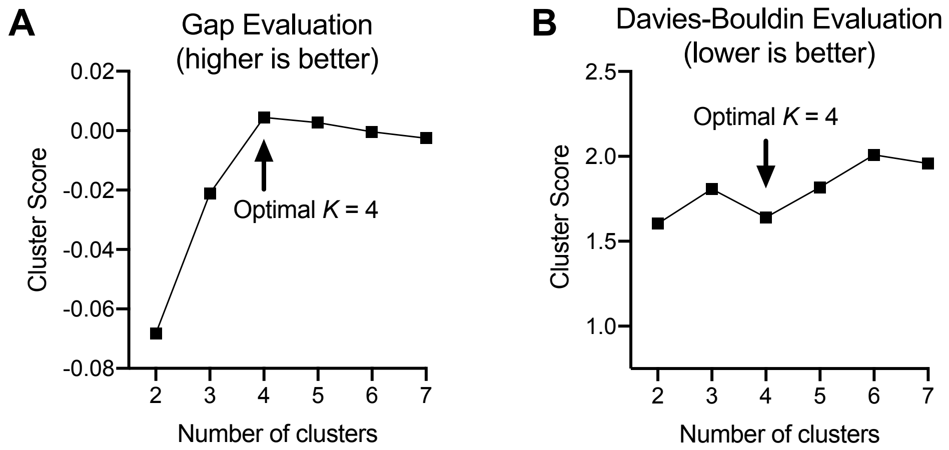

Supplementary figure 4

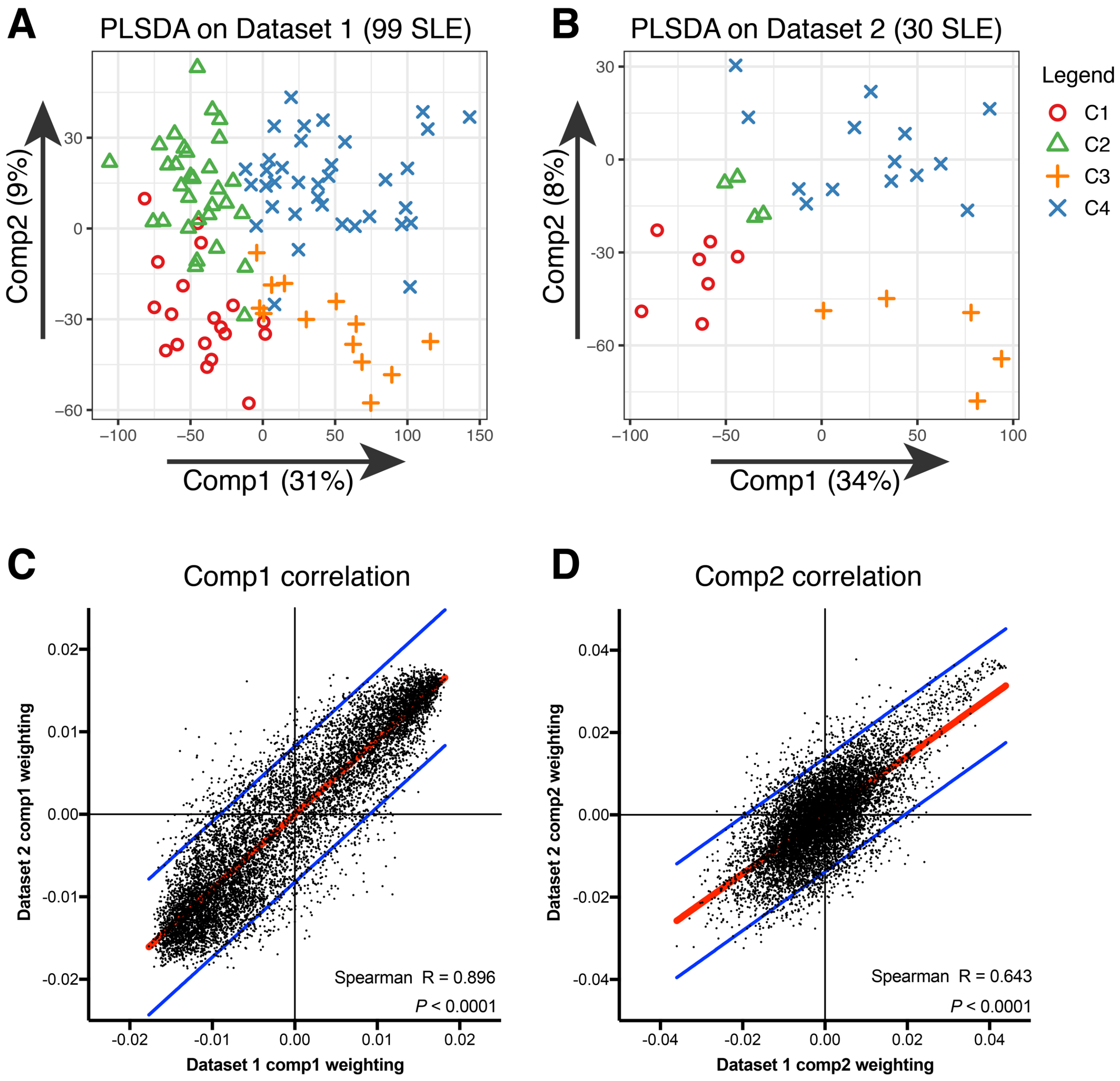

Supplementary figure 5

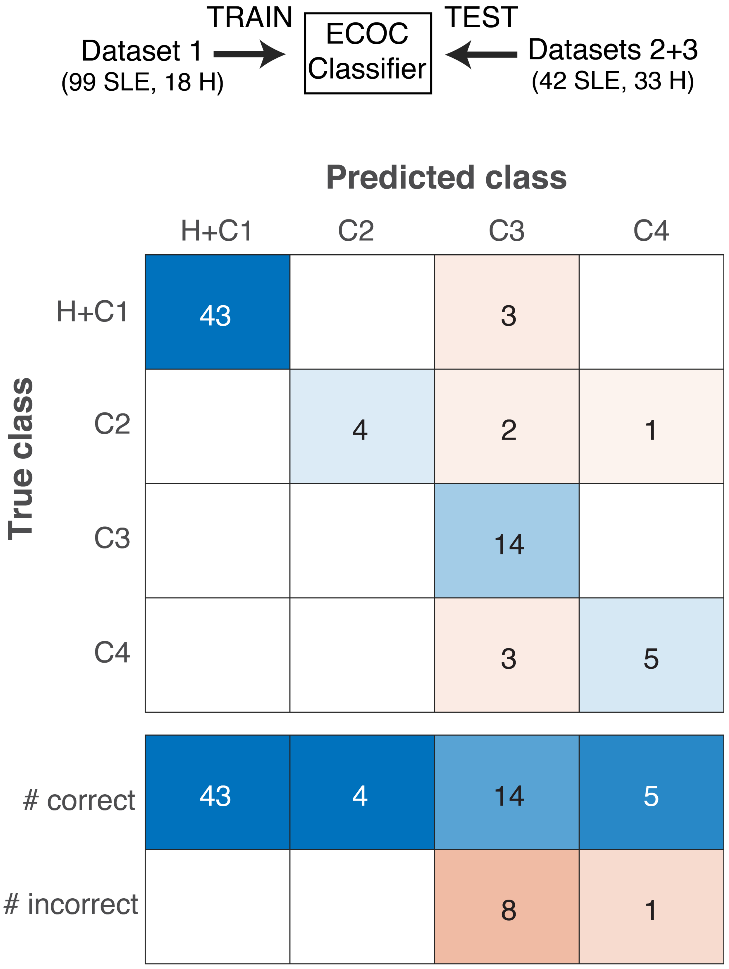

Overall: 66 / 75 (88%) correct classifications

Supplementary figure 6

A

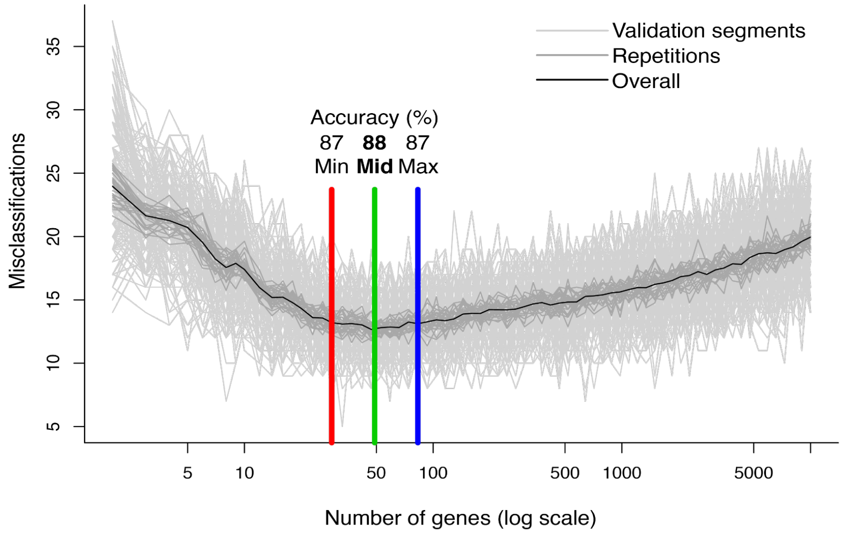

B

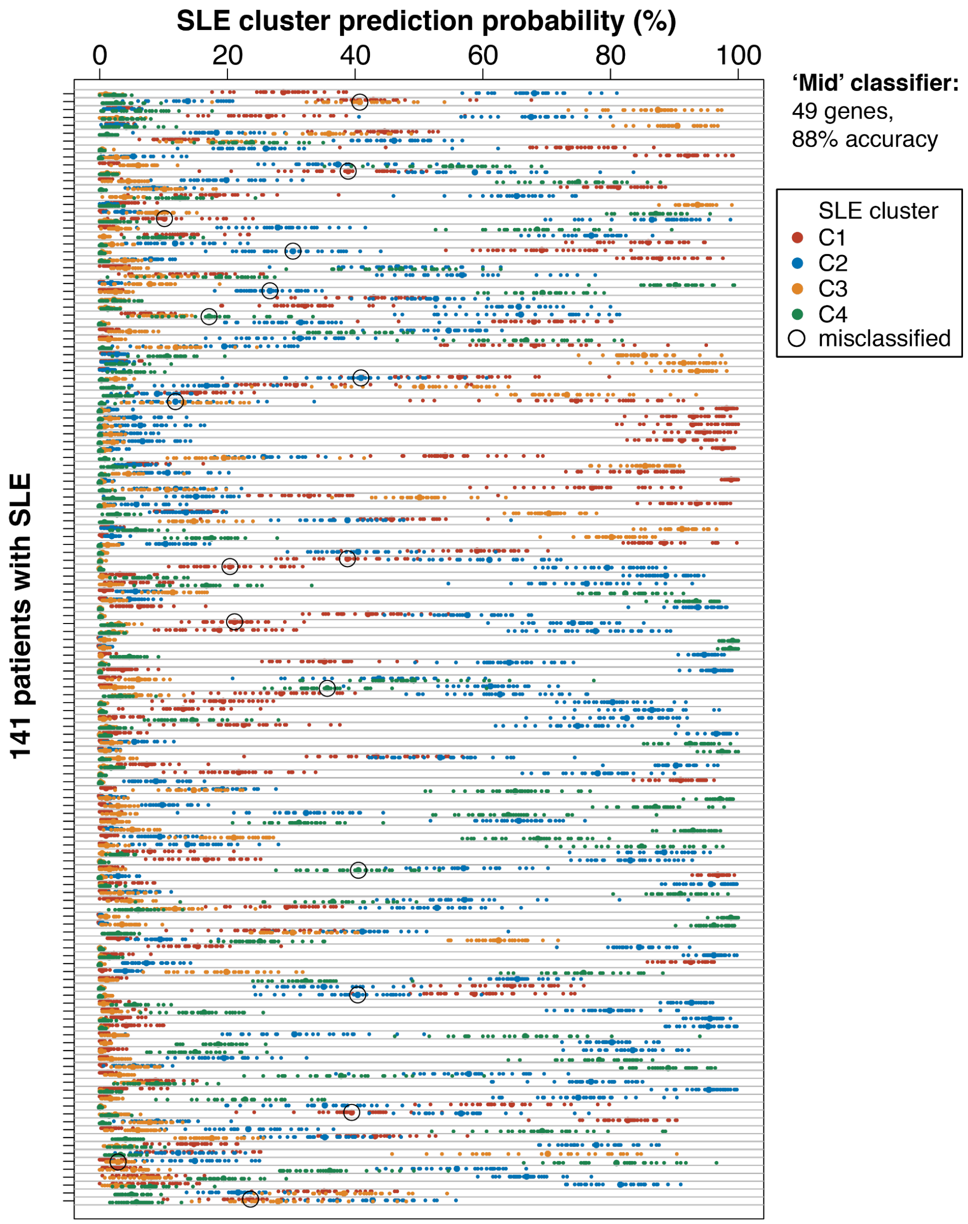

Supplementary figure 7

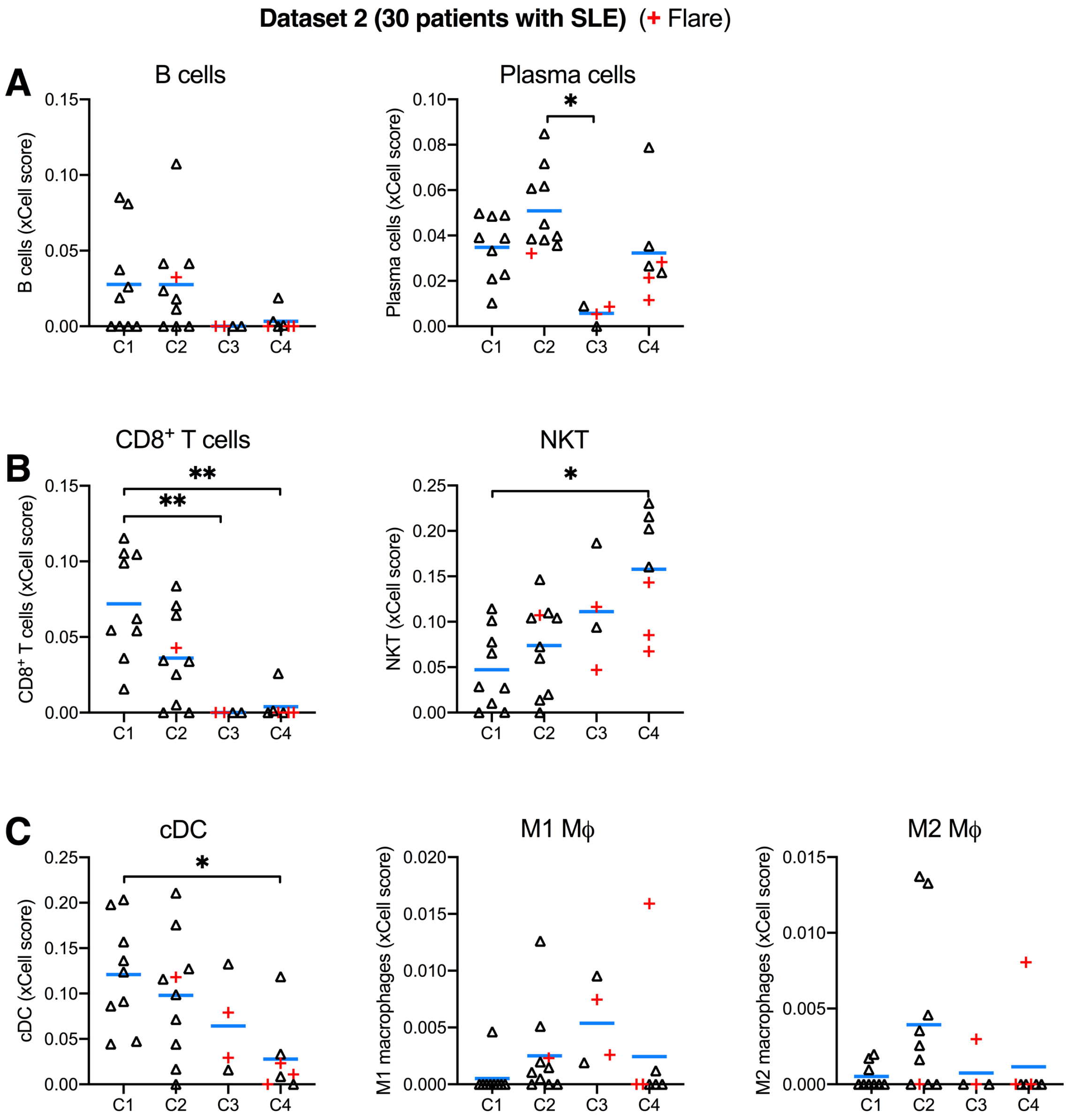

Supplementary figure 8

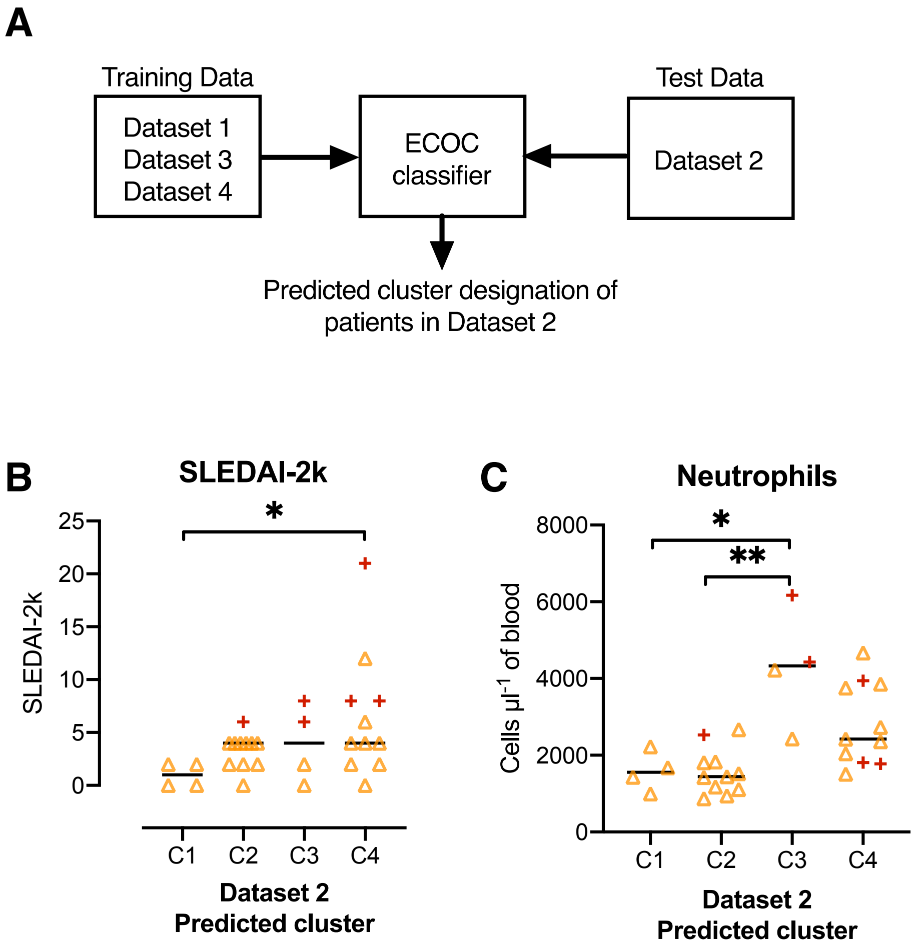

Supplementary figure 9

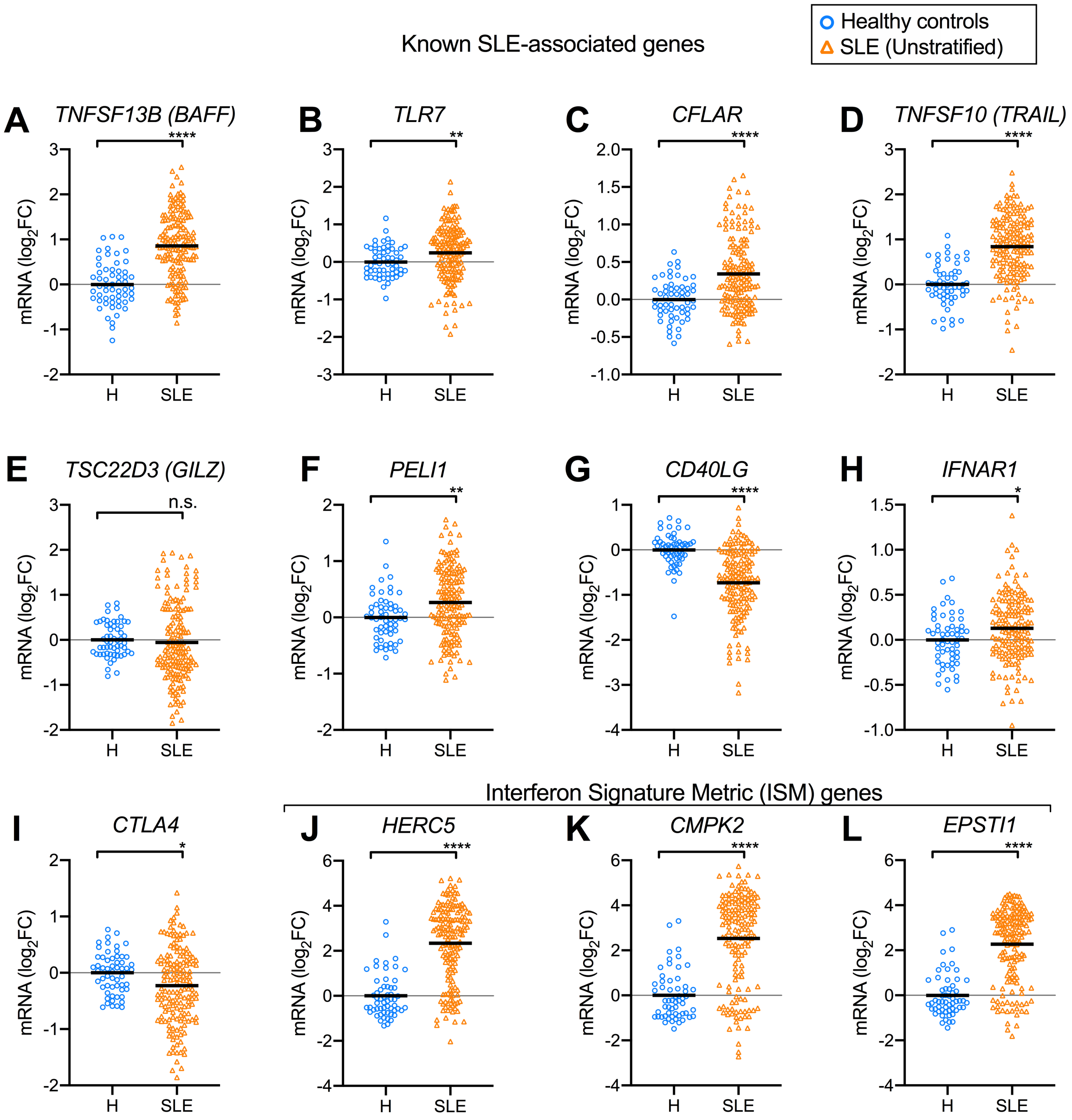

Supplementary figure 10

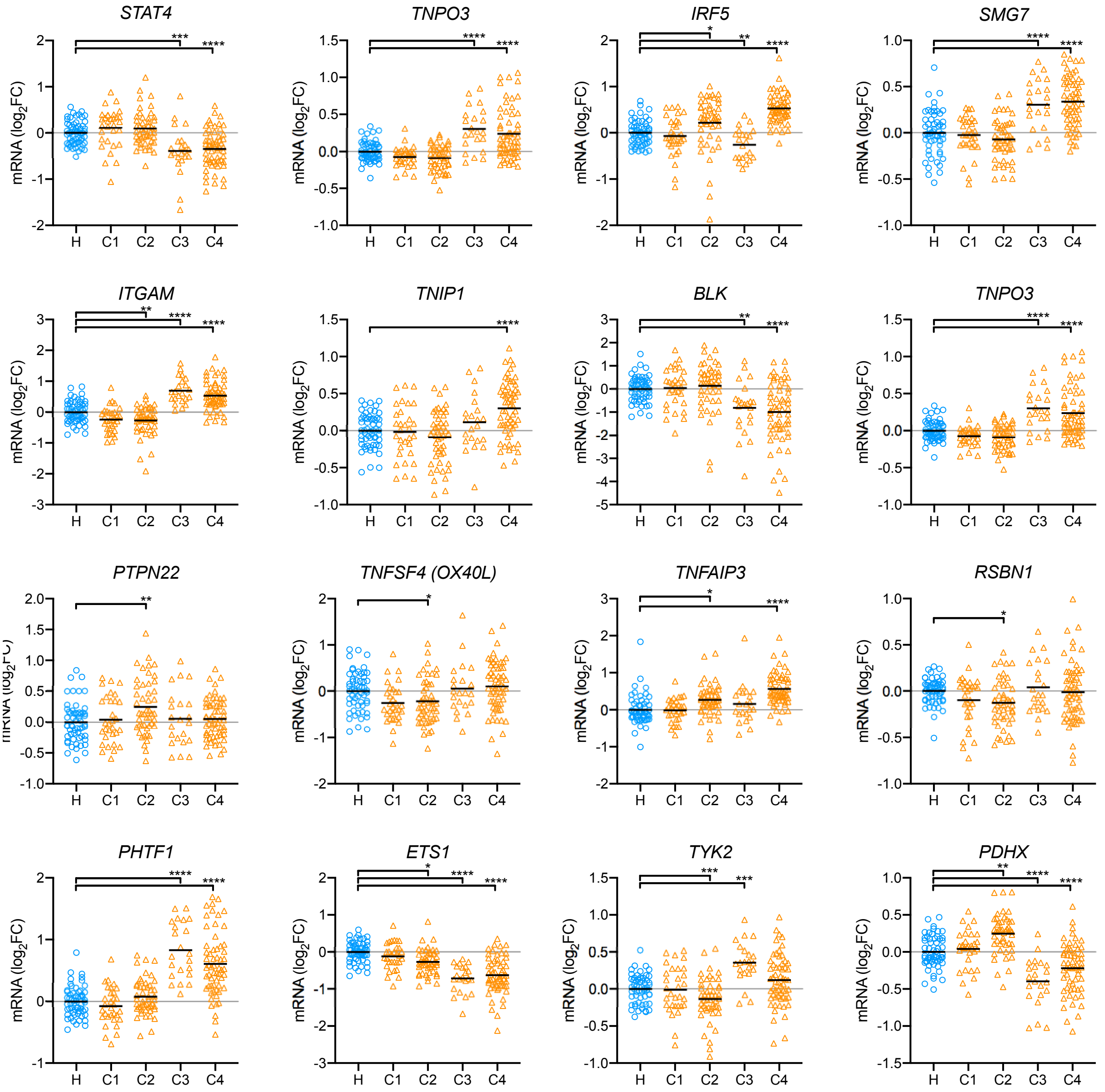

Supplementary figure 11

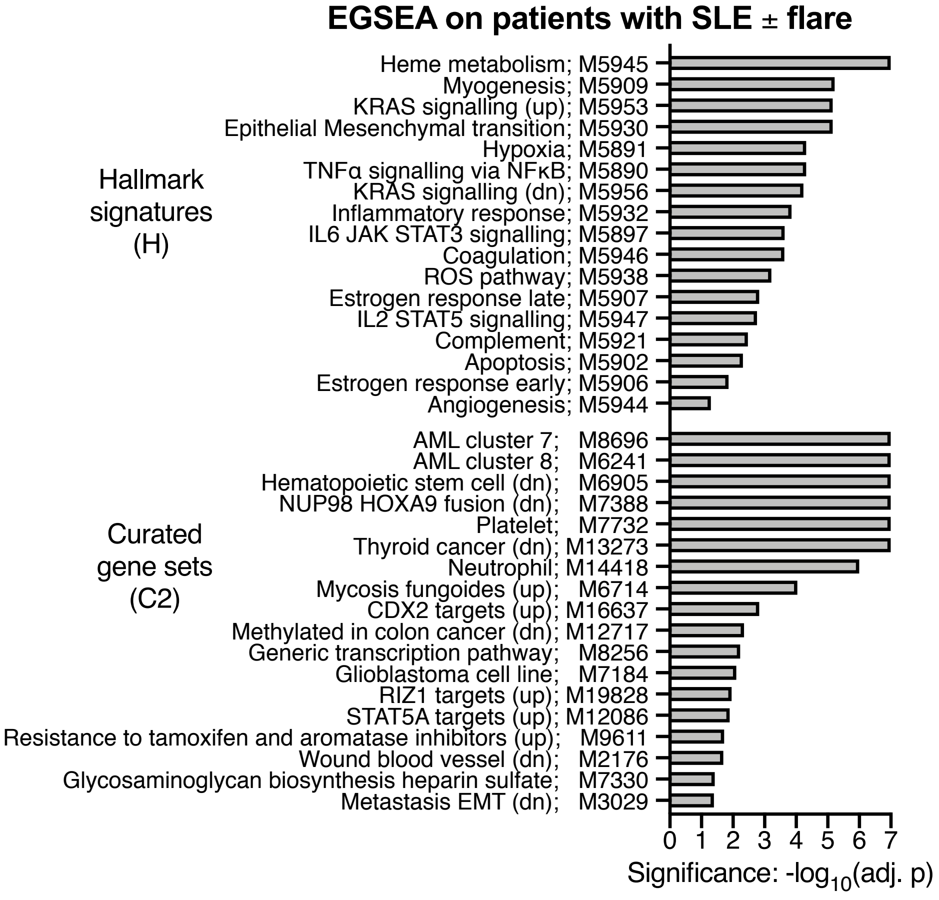

Supplementary figure 12

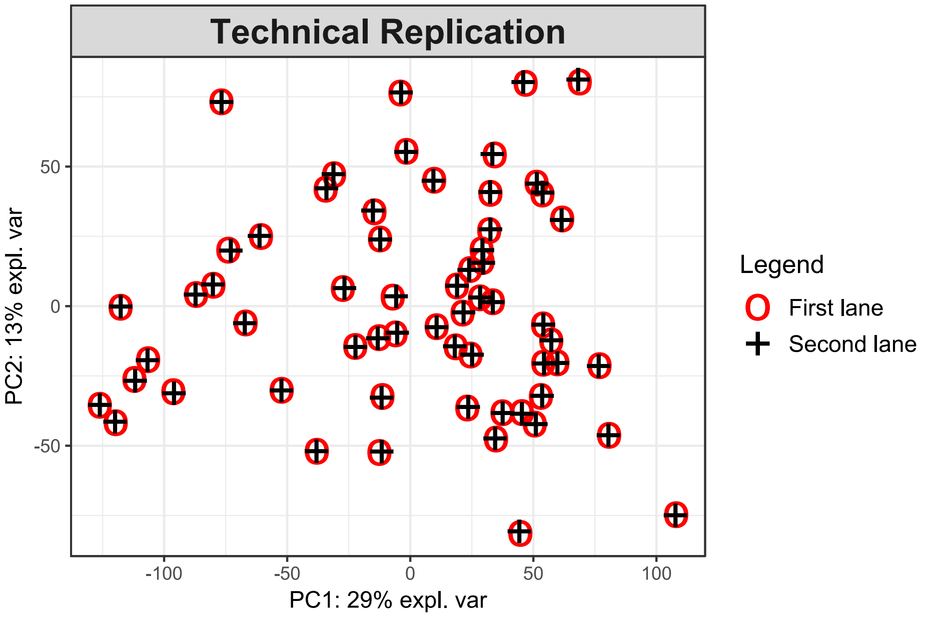

Supplementary table 1. Software used for processing RNA-seq data.

Software	Version	Purpose in this project	Ref. / Company
Ballgown	2.12.0	Calculate transcript abundance (FPKM).	[51]
BatchQC	1.10.1	Assessment and reduction of batch effects (ComBat).	[64]
edgeR	3.24.3	Count-based differential expression analysis.	[54]
EGSEA	1.10.1	Gene set enrichment analysis.	[59]
HISAT2	2.1.0	Gapped read alignment.	[51]
limma	3.38.3	Count-based differential expression analysis.	[54]
MATLAB	2018b	Clustering, machine learning.	MathWorks
mixOmics	6.6.1	Multivariate methods, variable selection.	[62]
MUVR	0.0.971	Variable selection, machine learning.	[57]
PRISM 8	8.0.2	Graphing and statistical tests.	GraphPad Software
R	3.5.2	Statistical programming.	[65]
R Studio	1.1.463	Integrated development environment for R.	[66]
SAMtools	1.8	Sorting read alignments.	[67]
SRA-toolkit	2.9.2	*fastq-dump*: Obtain archived fastq data.	[63]
Stringtie	1.3.5	Transcript/splice model assembly.	[51]
Subread	1.6.3	*featureCounts*: summarise read counts at the gene level.	[52]
Trimmomatic	0.38	mRNA read trimming.	[50]
xCell	1.1.0	Cell-type enrichment analysis.	[19]
